# Predictive Ability of the National Early Warning Score in Mortality Prediction of Acute Pulmonary Embolism in the Southeast Asian Population

**DOI:** 10.3390/jcdd10020060

**Published:** 2023-02-01

**Authors:** Chaiwat Bumroongkit, Pattraporn Tajarernmuang, Konlawij Trongtrakul, Chalerm Liwsrisakun, Athavudh Deesomchok, Chaicharn Pothirat, Theerakorn Theerakittikul, Atikun Limsukon, Nutchanok Niyatiwatchanchai, Juthamas Inchai, Warawut Chaiwong

**Affiliations:** Division of Pulmonary, Critical Care, and Allergy, Department of Internal Medicine, Faculty of Medicine, Chiang Mai University, Chiang Mai 50200, Thailand

**Keywords:** acute pulmonary embolism, mortality, national early warning score, NEWS

## Abstract

Background: The National Early Warning Scores (NEWS) easily and objectively measures acute clinical deterioration. However, the performance of NEWS to predict mortality in patients with acute pulmonary embolism (APE) is still required. Therefore, the objective of this study was to evaluate the performance of the NEWS in predicting the mortality of patients with APE. Methods: NEWS and Pulmonary Embolism Severity Index (PESI) at diagnosis time were calculated. Risk regression analysis was performed to identify the NEWS and PESI risk classification as a predictor for 30 days all-cause mortality and PE-related mortality. Results: NEWS was significantly higher in non-survivors compared to survivors (median (IQR) was 10 (7, 11) vs. 7 (2, 9), respectively, *p* < 0.001). The best cut-off point of NEWS in discriminating APE patients who non-survived from those who survived at 30 days was ≥9, with a sensitivity and specificity of 66.9% and 66.3%, respectively. The adjusted risk ratio of 30-day all-cause mortality in patients with initial NEWS ≥ 9 was 2.96 (95% CI; 2.13, 4.12, *p* < 0.001). Conclusions: The NEWS can be used for mortality prediction in patients with APE. APE patients with NEWS ≥ 9 are associated with a high risk of mortality and should be closely monitored.

## 1. Introduction

Venous thromboembolism (VTE) is one of the top three acute cardiovascular events [1]. Even though guidelines for improving the effectiveness of the diagnosis and management have been established, acute pulmonary embolism (APE) remains the major cause of cardiovascular mortality thus far [2,3]. APE can be presented in a broad range of clinical presentations from asymptomatic incidental findings on chest computerized tomography (CT) to severe hemodynamic instability, cardiovascular collapse, and sudden death. Several clinical prediction rules such as the Pulmonary Embolism Severity Index (PESI) score [4,5,6], simplified PESI (sPESI) score [7,8] and European Society of Cardiology (ESC) 2019 risk stratification [2] have been specifically proposed to assess the risk of early adverse outcomes, especially 30-day mortality after APE diagnosis. These scores can be used in triaging and management decisions of APE patients [2,3,5].

The National Early Warning Scores (NEWS) has been generated since 2012 and has been updated since 2017 as NEWS2 by the Royal College of Physicians of London [9,10]. The NEWS consists of six basic physiologic parameters, including heart rate (HR), systolic blood pressure (SBP), body temperature (BT), respiratory rate (RR), peripheral capillary oxygen saturation (SpO_2_), need for oxygen supplementation and level of consciousness. Since NEWS is easily and objectively measured by physicians and nurses, it is generally used in the emergency department (ED) and in hospital wards to detect acute clinical deterioration. The range of scores is 0–20. NEWS classifies acutely ill patients into three risk classes: low risk, medium risk, and high risk (aggregate score 0–4, 5–6, and ≥7, respectively). The higher the score, the higher the risk of intensive care unit (ICU) admission and poor clinical outcomes [9,10,11]. Several studies reported good predictive performance of NEWS in patients with sepsis [12,13,14].

A recent publication from Turkey demonstrated good predictive ability of NEWS and NEWS2 to predict 1-week mortality in patient with APE [15]. They found high areas under the curve (AUC), sensitivity, and specificity of cutoff NEWS ≥ 7.5 and NEWS2 ≥ 5.5 for 1-week mortality prediction (AUC = 0.854 (95% confidence interval (CI); 0.807, 0.902), sensitivity = 78%, and specificity = 73% and AUC = 0.870 (95% CI; 0.825, 0.915), sensitivity = 83%, and specificity = 73%, respectively). Another publication from Netherlands also showed that NEWS was a good predictor for ICU admission and 30-day mortality with an area under the receiver operating characteristic (AUROC) of 0.80 (95% CI; 0.66, 0.94) and 0.92 (95% CI; 0.82, 1.00), respectively [16]. They suggested that a threshold of NEWS ≥ 3 points yielded a sensitivity and specificity of 92% and 53% for ICU admission and 100% and 52% for 30-day mortality. However, the performance of NEWS to predict mortality in patients with APE has never been reported in the Southeast Asian population. The studies about the use of the NEWS for predicting mortality in the Asian population, especially the Thai population, are still required. Therefore, this study aimed to evaluate the discriminative ability of NEWS in predicting early mortality and comparing the performance of the prediction with PESI of hospitalized APE patients.

## 2. Materials and Methods

We performed a secondary analysis from the ten-year cross-sectional study, registry data of patients with APE in Maharaj Nakorn Chiang Mai Hospital, Faculty of Medicine, Chiang Mai University, a tertiary care, referral center located in Northern Thailand between January 2011 and December 2020 [17]. All Asian adults, in-patients, aged ≥15 years, diagnosed with APE by International Classification of Diseases 10th Revision (ICD-10) coding I26.0 (pulmonary embolism with acute cor pulmonale) and I26.9 (pulmonary embolism without acute cor pulmonale) were recruited into the study. All patient imaging was reviewed to confirm a diagnosis of APE. Diagnosis of APE was made when one of the radiological findings was met and confirmed by the radiologist. The radiologic criteria included (1) a demonstration of thrombus in the pulmonary artery and its branches by computed tomography pulmonary angiography (CTPA) or (2) a demonstration of thrombus in the pulmonary artery and its branches by chest CT with contrast.

The patient’s demographic data including signs, symptoms, risk factors, comorbidities, chest radiography, room air SpO_2_, cardiac troponin-T level, D-dimer level, electrocardiography, echocardiography, and 30-day outcome were reviewed from electronic medical records. For the patient with symptoms and signs of APE visited at ED, the clinical predictive scores including NEWS and PESI score were collected at the ED arrival time. For the inpatients with symptoms and signs of APE, the clinical parameters were collected close to the time of suspected APE. For incidental APE by CT chest for other reasons, the parameters were collected at the time that CT was requested. We excluded subjects with missing data required for calculating the clinical predictability scores (complete case analysis), old and repeated cases records, non-Asian ethnics, chronic thromboembolic pulmonary hypertension (CTEPH) cases, and others. The outcomes of interest were 30-day all-cause mortality and PE-related mortality after APE diagnosis. The study was approved by the Research Ethics Committee of the Faculty of Medicine, Chiang Mai University, Thailand approval number: MED-2564-08294).

### Statistical Analysis

Categorical variables were presented as numbers and percentages, while continuous variables were presented as the mean and standard deviation (SD) or the median and interquartile range (IQR) based on their distributions. Chi-Square test or Fisher’s exact test was used to compare categorical data, while Student’s *t* test or Mann–Whitney U test was used for comparing continuous variables as appropriate. To determine the discriminative ability of NEWS to predict 30-day all-cause and PE-related mortality, we calculated the AUROC curves, a plot of true positive rate (sensitivity) against false positive rate (1—specificity) for each scale level of NEWS to dichotomous outcomes of interest (non-survivor and survivor). Contingency tables were made to demonstrate the following performance, including sensitivity, specificity, positive likelihood ratio (+LR), negative likelihood ratio (−LR), and AUROC. The 95% confidence intervals (CI) for sensitivity, specificity, +LR, −LR, and AUROC were also provided. We identified the optimum cut-off point NEWS score in predicting mortality, using the Youden Index. Additionally, we plotted the Kaplan–Meier curve according to the best optimal NEWS and calculated the significant level using the log-rank test. Multivariable risk regression was performed to identify the NEWS and the PESI risk classification as a predictor for all-cause mortality and PE-related mortality at 30 days when adjusted for immobilization status. Results were displayed as adjusted risks ratio (RR) together with a 95% CI for RR. *p* value < 0.05 was considered statistically significant. All statistical analyses were performed using STATA version 16 (Stata Corp, College Station, TX, USA).

## 3. Results

**Figure 1:**
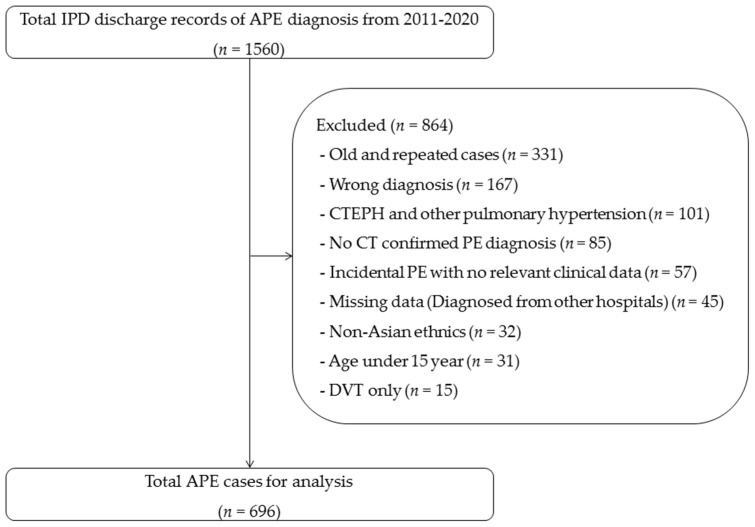
Study flow diagram. Abbreviations: IPD, inpatient department; APE, acute pulmonary embolism; CT, computerized tomography; DVT, deep vein thrombosis; CTEPH, chronic thromboembolic pulmonary hypertension.

There were 1560 inpatient discharge records of APE diagnosis from 2011–2020. After exclusion of 864 subjects due to old and repeated cases (*n* = 331), wrong diagnosis (*n* = 167), CTEPH and other pulmonary hypertension (*n* = 101), no computed tomography (CT) confirmed PE diagnosis (*n* = 85), incidental PE with no relevant clinical data (*n* = 57), missing data (diagnosed from other hospitals) (*n* = 45), non-Asian ethnics (*n* = 32), age under 15 year (*n* = 31), and DVT only (*n* = 15), a total of 696 patients with new APE diagnoses were enrolled in the study. The study flow diagram is shown in Figure 1. Among these, the diagnosis of APE was based on CTPA for 468 patients and CT chest with contrast for 228 patients. Among these, 133 (19.1%) patients were classified as death from any cause within 30 days, and 39 (5.6%) patients died due to PE. The proportion of male and active malignancy was significantly greater in the non-survivors while incidental PE and unprovoked (idiopathic) PE had a greater proportion in the survivors. The non-survivors had more symptoms and signs including dyspnea, chest pain, tachycardia, hypotension, desaturation, and acute respiratory failure than the survivors. Systolic and diastolic blood pressures were significantly lower in the non-survivors group. There were no statistical differences in right ventricle (RV) strain pattern from ECG, RV dysfunction from echocardiography, and RV/left ventricle (LV) >1 from computed tomography imaging between groups. However, the level of serum biomarkers including troponin-T and D-dimer were significantly higher in the non-survivors group than in the survivors. Non-survivors had significantly higher PESI with a median (IQR) of 133 (107, 153) vs. 101 (85,121) and a higher NEWS score of 10 (7, 11) vs. 7 (2, 9) than survivors. More data are presented in Table 1.

The discriminative property of each NEWS cut-off point for 30-day all-cause mortality prediction is shown in Table 2. The best cut-off value for the NEWS score was ≥9.0 with a sensitivity of 66.9% (95% CI; 58.2, 74.8), specificity of 66.3% (95% CI; 62.2, 70.2), positive likelihood ratio of 1.98 (95% CI; 1.68, 2.34), negative likelihood ratio of 0.50 (95% CI; 0.39, 0.64), and an AUROC of 0.67 (95% CI; 0.62, 0.71). The discriminative property of each NEWS cut-off point for 30-day PE-related mortality prediction is also shown in Table 3. The cut-off value for the NEWS score was ≥11.0 with a sensitivity of 76.9% (95% CI; 60.7, 88.9), specificity of 85.8% (95% CI; 82.9, 88.4), positive likelihood ratio of 5.43 (95% CI; 4.21, 7.01), negative likelihood ratio of 0.27 (95% CI; 0.15, 0.48), and an AUROC of 0.81 (95% CI; 0.75, 0.88).

The proportions of deceased patients (all-cause and PE-related mortality) divided by NEWS, PESI classification, and the adjusted risk ratio of 30-day mortality for each category is presented in Table 4. After adjusting by immobilized status, the adjusted risk of all-cause mortality in 30 days in the high-risk NEWS class (≥7) was 2.3-fold higher than the low-risk NEWS class (0–4) (adjusted risks ratio: 2.33 (95% CI; 1.54, 3.53), *p* < 0.001). The adjusted risk of 30-day all-cause mortality at the best cut-off point of NEWS (≥9) was 2.96-fold higher than NEWS < 9 (adjusted risks ratio: 2.96 (95% CI; 2.13, 4.12), *p* < 0.001). The adjusted risk of all-cause mortality in 30 days in the PESI class V was 6.0-fold higher than the PESI class I–II (adjusted risks ratio: 6.00 (95% CI; 3.21, 11.24), *p* < 0.001). The adjusted risk of PE-related mortality in 1 month of NEWS ≥ 9 was 13.1-fold higher than NEWS < 9 (adjusted risks ratio: 13.14 (95% CI; 4.72, 36.56), *p* < 0.001). The adjusted risk of PE-related mortality in 30 days in the high-risk NEWS class was 21.9-fold higher than the low-risk NEWS class (adjusted risks ratio: 21.87 (95% CI; 3.02, 158.35), *p* < 0.001). The adjusted risk of PE-related mortality in 30 days in the PESI class V was 27.8-fold higher than the PESI class I–II (adjusted risks ratio: 27.84 (95% CI; 3.85, 201.36), *p* = 0.001). More data are shown in Table 4.

**Figure 2:**
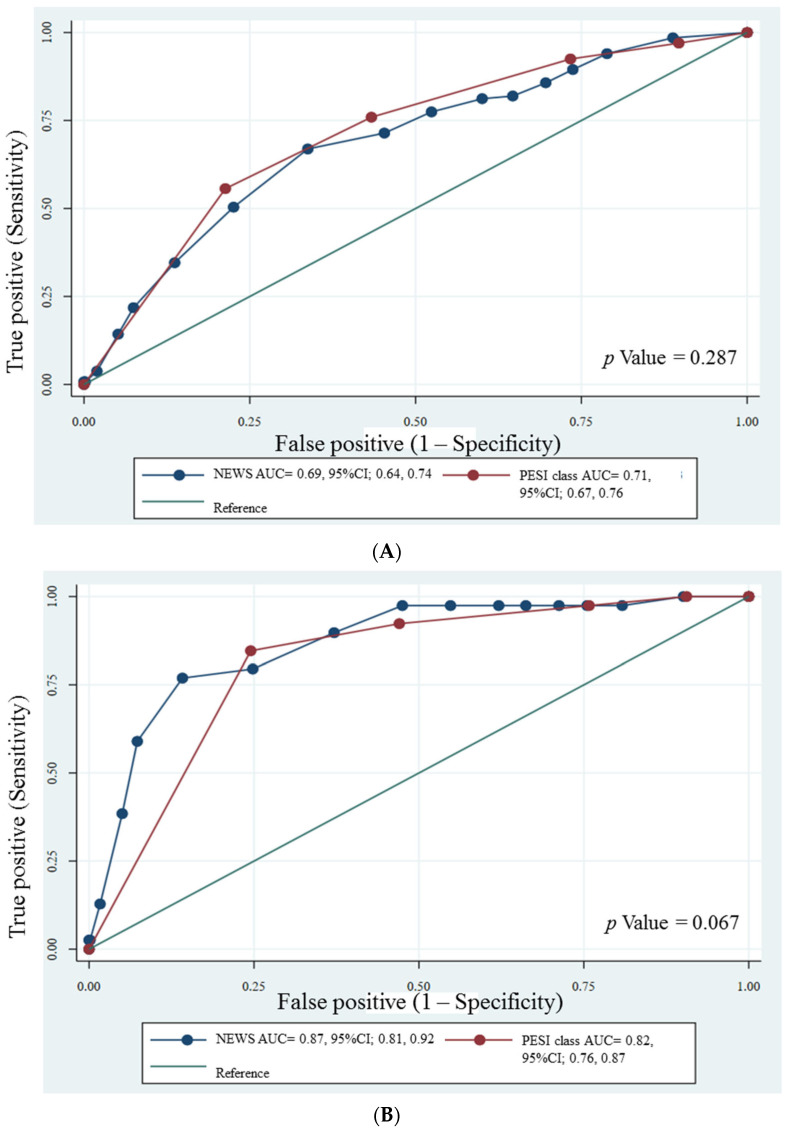
Receiver operating curve of the NEWS and PESI classifications for 30-day all-cause mortality (**A**) and PE-related mortality (**B**) prediction in patients with acute pulmonary embolism. Abbreviations: NEWS, National Early Warning Score; PESI, Pulmonary Embolism Severity Index.

The AUROC curve of the NEWS and PESI classifications to predict 30-day all-cause mortality and 30-day PE-related mortality are shown in Figure 2A,B, respectively. The AUROC values of NEWS and PESI in predicting 30-day all-cause mortality were 0.69 (95% CI; 0.64, 0.74) and 0.71 (95% CI; 0.67, 0.76), respectively. The AUROC values of NEWS and PESI classifications in predicting 30-day PE-related mortality were 0.87 (95% CI; 0.81, 0.92) and 0.82 (95% CI; 0.76, 0.87), respectively. The AUROC of NEWS and PESI for predicting 30-day all-cause mortality and PE-related mortality did not differ significantly.

**Figure 3:**
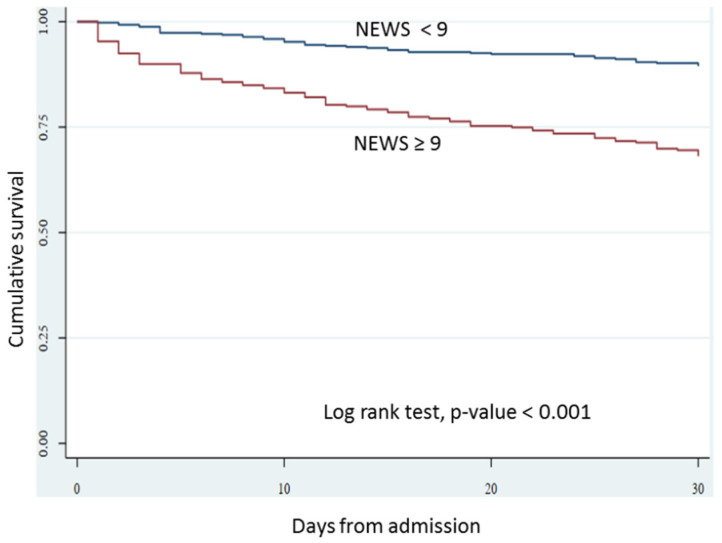
Survival plot of 30-day all-cause mortality in APE patients according to NEWS. Abbreviations: NEWS, National Early Warning Score.

The Kaplan–Meier survival plots of 30-day all-cause mortality in APE patients according to NEWS are shown in Figure 3. There was significantly higher 30-day mortality in patients with the high NEWS group (≥9) versus the low NEWS group (<9); log-rank test, *p* < 0.001.

## 4. Discussion

The National Early Warning Score (NEWS) and the updated version in 2017, NEWS2, has been widely used and validated in several clinical settings, including in ED settings and in general wards. The discriminative ability of NEWS in determining the mortality outcome has been studied in patients with community-acquired pneumonia (CAP). The presence of a high NEWS score in a patient who was admitted to the ED with CAP was associated with the risk of 30-day mortality [18]. In addition, the increased NEWS can predict 90-day mortality in acutely dyspneic patients who visited ED [19]. Of these, 3% of the patients were diagnosed with APE. Several studies demonstrated that the use of NEWS can predict poor outcomes in patients with sepsis better than the other scoring systems, such as systemic inflammatory response syndrome (SIRS) or quick Sepsis-Related Organ Failure Assessment (qSOFA) [12,20]. In the COVID-19 infection study, Jang et al. found that the NEWS score was superior to qSOFA and SIRS in mortality prediction [21].

There are many specific rules for predicting mortality in PE and PESI, and a simplified version of sPESI shows the most validated specific rules. We mention comparing the NEWS, which a general rule, to the previously validated specific rule. The ideal tool for severity and mortality prediction should be easy to apply, bedside applicable (calculation), non-laboratory dependent, and non-sophisticated test dependent, with no historical data required. Recently, Yolcu et al. found that NEWS ≥ 7.5 and NEWS2 ≥ 5.5 had good ability to predict 1-week mortality in patients with APE with a high AUROC, sensitivity, and specificity [15]. Other post hoc analyses [16] of the YEARS study [22] compared the predictive performance of NEWS, PESI, and sPESI for predicting 7-day ICU admission and 30-day mortality in patients with hemodynamically stable APE. The study demonstrated that NEWS ≥ 3 had greater predictive ability than PESI and sPESI to predict 7-day ICU admission, and NEWS ≥ 7 showed comparable predictive ability for 30-day all-cause mortality [16].

Regarding the results of our study, we found an optimal discriminative ability of NEWS in terms of determining the 30-day all-cause mortality in APE patients (AUROC = 0.69 (95% CI; 0.64, 0.74)). However, the predicting ability of NEWS was more accurate in PE-related mortality (AUROC = 0.87 (95% CI; 0.81–0.92)). For a more thorough examination, we found higher prevalence of tachycardia, tachypnea, hypotension, and desaturation in the non-survivor group. These features resulted in a higher NEWS score in the non-survivors group than for those who survived. Additionally, an accumulated NEWS score of ≥9 increased the risk of death approximately 3-fold for 30-day all-cause mortality and 13-fold for 30-day PE-related mortality. When using the NEWS threshold of ≥7 (high-risk) that was recommended by developers, we found that high-risk NEWS will increase 2.33-fold for 30-day risk of all-cause death and 21.87-fold for 30-day risk of PE-related mortality, which is comparable to the risk of death in PESI classes IV–V. Nevertheless, the cut-off value of NEWS at ≥ 9 in our study was superior in predicting 30-day all-cause mortality.

The 30-day all-cause mortality of patients with APE in our study (19.1%) was higher than previous reports (5.9%), and for the patients with PESI class V, it was 38% vs. 17.9% [6]. Another recent study in normotensive elderly patients showed overall mortality of less than 5% [23]. One meta-analysis published in 2016 also showed the overall 30-day mortality of 11.4% (9.9–13.1%) in the high-risk PESI group [24]. These findings can be explained form the higher proportion of patients with active malignancy in our study (55%) than in the previous studies (9–21%); in addition, the proportions of active DVT and recent surgery were higher than in the previous reports [6,23]. Nevertheless, the data on the actual prevalence of venous thromboembolism including DVT and APE in the Southeast Asian population including Thailand are lacking. The high mortality of patients with APE from this retrospective cohort should raise awareness of physicians to prompt diagnosis and provide appropriate risk stratification and treatment. This study demonstrated the usefulness of NEWS to assess patients with APE, since it is an easy tool and is already widely used in many emergency departments and general wards.

There are some limitations in this study. Firstly, the PE-related mortality might be underestimated because all PE-related death occurred in-hospital. The patients who died after discharge from the hospital within 30 days were counted in all-cause mortality. Secondly, arterial blood gas (ABG) analysis was not performed in every patient; we estimated the arterial oxygen saturation for PESI by using SpO_2_. Thirdly, the proportion of chronic obstructive pulmonary disease (COPD) patients was small and showed no difference between survivors and non-survivors. Thus, we calculated the score of SpO2 using the original NEWS in COPD patients. Moreover, the risk of selection bias is unavoidable due to retrospective data collection of this study. For example, only hospitalized patients were included. There was a possibility of false-positive APE in selected patients due to one-third of APE diagnoses being made with chest CT with contrast, which showed lower diagnostic performances than CTPA, where the assessment criteria were based on medical records. Although this study enrolled only Asian ethnicities, we believed that our study results could apply to using NEWS in predicting the mortality of APE without an ethnic difference. There were several changes in the guidelines of management during the study period, such as the 2014 ESC and 2019 ESC guidelines. We did not examine whether physicians’ practices had changed, which might have affect the outcome.

## 5. Conclusions

Our retrospective trial suggested that NEWS could predict mortality in patients with APE and that ≥9 could be an appropriate cutoff to define high-risk patients requiring monitoring. However, these results should be confirmed in future prospective studies.

## Figures and Tables

**Table 1:** Baseline characteristics of patients with acute pulmonary embolism and survival status at 30 days after diagnosis.

Characteristics	All Patients (*n* = 696)	Non-Survivors (*n* = 133)	Survivors (*n* = 563)	*p* Value
Age (years)	57.7 ± 15.7	59.2 ± 14.3	57.4 ± 15.9	0.228
Male, *n* (%)	286 (41.1)	67 (50.4)	219 (38.9)	0.019
Comorbidities				
Hypertension	305 (43.8)	54 (40.6)	251 (44.6)	0.438
Diabetes mellitus	114 (16.4)	23 (17.3)	91 (16.2)	0.419
Heart failure	42 (6.0)	8 (6.0)	34 (6.0)	1.000
COPD	38 (5.5)	11 (8.3)	27 (4.8)	0.135
Cirrhosis	31 (4.5)	10 (7.5)	21 (3.7)	0.064
Obesity	12 (1.7)	2 (1.5)	10 (1.8)	1.000
Risk factors				
Active malignancy	383 (55.0)	100 (75.2)	283 (50.4)	<0.001
Surgery (<4 weeks)	141 (20.3)	21 (15.8)	120 (21.4)	0.187
Immobilization	211 (30.4)	50 (37.6)	161 (28.6)	0.047
Long travel	10 (1.4)	1 (0.8)	9 (1.6)	0.696
Unprovoked (idiopathic) PE	136 (19.5)	13 (9.8)	123 (21.8)	0.004
Active DVT	245 (35.2)	51 (38.3)	194 (34.5)	0.674
Symptoms and signs				
Asymptomatic (incidental) PE	228 (32.8)	29 (21.8)	199 (35.4)	0.003
Dyspnea	453 (65.1)	109 (82.0)	344 (61.3)	<0.001
Chest pain	112 (16.1)	14 (10.5)	98 (17.5)	0.018
Syncope	54 (7.8)	14 (10.5)	40 (7.1)	0.207
Heart rate (bpm)	105.4 ± 18.4	112.5 ± 17.7	103.6 ± 18.1	<0.001
Tachycardia (HR ≥ 110/min)	326 (46.8)	89 (66.9)	237 (42.2)	<0.001
Respiratory rate (bpm)	24.3 ± 6.1	26.7 ± 6.6	23.7 ± 65.9	<0.001
Tachypnea (RR ≥ 30/min)	158 (22.7)	51 (38.3)	107 (19.0)	<0.001
SBP (mmHg), mean ± SD	116.4 ± 21.9	110.4 ± 22.2	117.8 ± 21.7	0.001
DBP (mmHg), mean ± SD	72.3 ± 13.9	68.9 ± 14.3	73.1 ± 13.7	0.002
MAP (mmHg), mean ± SD	87.0 ± 15.7	82.8 ± 16.2	88.0 ± 15.4	0.001
SpO_2_ (%)	89.4 ± 7.9	86.9 ± 7.4	89.9 ± 8.0	<0.001
Desaturation (SpO_2_ < 90%)	333 (47.8)	89 (67.4)	244 (43.8)	<0.001
Acute respiratory failure *	129 (18.5)	48 (36.1)	81 (14.4)	<0.001
Electrocardiogram				
Sinus tachycardia	393 (56.5)	101 (75.9)	292 (51.9)	<0.001
S1Q3T3	160 (23.0)	35 (26.3)	125 (22.2)	0.470
RV strain pattern	112 (16.1)	21 (15.8)	91 (16.2)	0.728
Echocardiography				
RV dysfunction (*n* = 332)	132 (39.8)	26 (42.6)	106 (39.1)	0.665
CT imaging				
RV/LV > 1	190 (27.3)	43 (32.3)	147 (26.1)	0.160
Mediastinal (central) emboli	310 (44.5)	51 (38.3)	259 (46.0)	0.121
Biomarkers				
Troponin-T, pg/mL (median, IQR) (*N* = 228)	53.1 (19.6, 159.5)	122.0 (34.0, 233.3)	40.2 (16.0, 114.5)	0.002
D-dimer, ng/mL (median, IQR) (*N* = 129)	8850.0 (4242.5, 18,748.5)	12,820.0 (7058.0, 32,909.0)	7316.0 (3898.0, 14,389.0)	0.024
Predictive scores				
NEWS2 (median, IQR)	8.0 (3.0, 10.0)	10.0 (7.0, 11.0)	7.0 (2.0, 9.0)	<0.001
PESI score (median, IQR)	106.0 (88.0, 129.0)	133.0 (107.0, 153.0)	101.0 (85.0, 121.0)	<0.001

Note: Data are presented as mean ± SD or *n* (%) and median, IQR for D-dimer, Troponin-T, NEWS, PESI.; *, respiratory failure defined as the requiring of intubation and mechanical ventilation. Abbreviations: BP, blood pressure; COPD, chronic obstructive pulmonary disease; CT, computed tomography; DVT, deep vein thrombosis; LV, left ventricle; NEWS, National Early Warning Score; OSA, obstructive sleep apnea; PE, pulmonary embolism; PESI, Pulmonary Embolism Severity Index Score RV, right ventricle; SpO_2_, pulse oximetry.

**Table 2:** Discriminative property of the NEWS cut-off points for predicting 30-day all-cause mortality in patients with acute pulmonary embolism.

NEWS Cut-Off	Sensitivity (%) (95% CI)	Specificity (%) (95% CI)	LR+ (95% CI)	LR− (95% CI)	AUROC (95% CI)
≥5.0	82.0 (74.4, 88.1)	35.3 (31.4, 39.5)	1.27 (1.15, 1.40)	0.51 (0.35, 0.75)	0.59 (0.55, 0.63)
≥6.0	81.2 (73.5, 87.5)	40.0 (35.9, 44.1)	1.35 (1.22, 1.50)	0.47 (0.33, 0.69)	0.61 (0.57, 0.65)
≥7.0	77.4 (69.4, 84.2)	47.6 (43.4, 51.8)	1.48 (1.31, 1.67)	0.47 (0.34, 0.66)	0.63 (0.58, 0.67)
≥8.0	71.4 (63.0, 78.9)	54.7 (50.5, 58.9)	1.58 (1.37, 1.82)	0.52 (0.39, 0.69)	0.63 (0.59, 0.67)
≥9.0	66.9 (58.2, 74.8)	66.3 (62.2, 70.2)	1.98 (1.68, 2.34)	0.50 (0.39, 0.64)	0.67 (0.62, 0.71)
≥10.0	50.4 (41.6, 59.2)	77.4 (73.8, 80.9)	2.23 (1.78, 2.80)	0.64 (0.54, 0.77)	0.64 (0.59, 0.69)
≥11.0	34.6 (26.6, 43.3)	86.3 (83.2, 89.1)	2.53 (1.85, 3.46)	0.76 (0.67, 0.86)	0.61 (0.56, 0.65)
≥12.0	21.8 (15.1, 29.8)	92.5 (90.0, 94.6)	2.92 (1.89, 4.51)	0.85 (0.77, 0.93)	0.57 (0.54, 0.61)
≥13.0	14.3 (8.8, 21.4)	94.8 (92.7, 96.5)	2.77 (1.61, 4.79)	0.90 (0.84, 0.97)	0.55 (0.51, 0.58)

Abbreviations: AUROC, area under receiver operating characteristic curve; NEWS, National Early Warning Score; LR+, positive likelihood ratio; LR−, negative likelihood ratio.

**Table 3:** Discriminative property of the NEWS cut-off points for 30-day PE-related mortality prediction in Asian population with acute pulmonary embolism.

NEWS Cut-Off	Sensitivity (%) (95% CI)	Specificity (%) (95% CI)	LR+ (95% CI)	LR− (95% CI)	AUROC (95% CI)
≥5.0	97.4 (86.5, 99.9)	33.8 (30.2, 37.5)	1.47 (1.37, 1.59)	0.08 (0.01, 0.53)	0.66 (0.63, 0.69)
≥6.0	97.4 (86.5, 99.9)	37.9(34.2, 41.7)	1.57 (1.45, 1.70)	0.07 (0.01, 0.47)	0.68 (0.65, 0.71)
≥7.0	97.4 (86.5, 99.9)	45.2 (41.4, 49.1)	1.78 (1.63, 1.94)	0.06 (0.01, 0.39)	0.71 (0.68, 0.75)
≥8.0	97.4 (86.5, 99.9)	52.5 (48.6, 56.4)	2.05 (1.87, 2.26)	0.05 (0.01, 0.34)	0.75 (0.72, 0.78)
≥9.0	89.7 (75.8, 97.1)	62.9 (59.0, 66.6)	2.42 (2.09, 2.79)	0.16 (0.06, 0.41)	0.76 (0.71, 0.82)
≥10.0	79.5 (63.5, 90.7)	75.2 (71.7, 78.4)	3.20 (2.60, 3.94)	0.27 (0.15, 0.51)	0.77 (0.71, 0.84)
≥11.0	76.9 (60.7, 88.9)	85.8 (82.9, 88.4)	5.43 (4.21, 7.01)	0.27 (0.15, 0.48)	0.81 (0.75, 0.88)
≥12.0	59.0 (42.1, 74.4)	92.7 (90.4, 94.6)	8.07 (5.53, 11.80)	0.44 (0.30, 0.65)	0.76 (0.68, 0.84)
≥13.0	38.5 (23.4, 55.4)	95.0 (93.0, 96.5)	7.66 (4.56, 12.90)	0.65 (0.51, 0.83)	0.67 (0.59, 0.75)

Abbreviations: AUROC, area under receiver operating characteristic curve; NEWS, National Early Warning Score; LR+, positive likelihood ratio; LR−, negative likelihood ratio.

**Table 4:** Thirty-day all-cause mortality and thirty-day PE-related mortality according to NEWS category and PESI classification.

Predictive Scores	All-Cause Death (*n* = 133)	Adjusted Risk Ratio * (95% CI)	*p* Value	PE-Related Death (*n* = 39)	Adjusted Risk Ratio * (95% CI)	*p* Value
**NEWS category**						
Low (0–4) (*n* = 223)	24 (18.1)	Ref.		1 (2.6)	Ref.	
Medium (5–6) (*n* = 75)	6 (4.5)	0.71 (0.30, 1.68)	0.440	0 (0.0)	N.A. *	
High (≥7) (*n* = 398)	103 (77.4)	2.33 (1.54, 3.53)	<0.001	38 (97.4)	21.87 (3.02, 158.35)	0.002
**NEWS** **cut off in this study**						
NEWS < 9 (*n* = 417)	44 (10.6)	Ref.		4 (1.0)	Ref.	
NEWS ≥ 9 (*n* = 279)	89 (31.9)	2.96 (2.13, 4.12)	<0.001	35 (12.5)	13.14 (4.72, 36.56)	<0.001
**PESI classification**						
I–II (*n* = 160)	10 (6.3)	Ref.		1 (0.6)	Ref.	
III (*n* = 191)	22 (11.5)	1.84 (0.89, 3.77)	0.096	2 (1.0)	1.67 (0.15, 18.31)	0.674
IV (*n* = 151)	27 (17.9)	2.86 (1.43, 5.70)	0.003	3 (2.0)	3.20 (0.34, 30.44)	0.311
V (*n* = 194)	74 (38.1)	6.00 (3.21, 11.24)	<0.001	33 (17.0)	27.84 (3.85, 201.36)	0.001

Note: N.A., no event; *, adjusted risks ratio; adjusted for immobilization status. Abbreviations: NEWS, National Early Warning Score; PESI, Pulmonary Embolism Severity Index.

## Data Availability

The data that support the findings of this study are available on request from the corresponding author.
